# Are alarm symptoms predictive of cancer survival? Population-based cohort study

**DOI:** 10.3399/bjgp13X675197

**Published:** 2013-11-06

**Authors:** Alex Dregan, Henrik Møller, Judith Charlton, Martin C Gulliford

**Affiliations:** King’s College London, Department of Primary Care and Public Health Sciences, London, and NIHR Biomedical Research Centre at Guy’s and St Thomas’ NHS Foundation Trust, London.; King’s College London, Division of Cancer Studies, Thames Cancer Registry, London.; King’s College London, Department of Primary Care and Public Health Sciences, London, and NIHR Biomedical Research Centre at Guy’s and St Thomas’ NHS Foundation Trust, London.; King’s College London, Department of Primary Care and Public Health Sciences, London, and NIHR Biomedical Research Centre at Guy’s and St Thomas’ NHS Foundation Trust, London.

**Keywords:** alarm symptom, colorectal cancer, gastro-oesophageal cancer, general practice, primary care, survival

## Abstract

**Background:**

Alarm symptom presentations are predictive of cancer diagnosis but may also be associated with cancer survival.

**Aim:**

To evaluate diagnostic time intervals, and consultation patterns after presentation with alarm symptoms, and their association with cancer diagnosis and survival.

**Design and setting:**

Cohort study using the Clinical Practice Research Database, with linked Cancer Registry data, in 158 general practices.

**Method:**

Participants included those with haematuria, haemoptysis, dysphagia, and rectal bleeding or urinary tract cancer, lung cancer, gastro-oesophageal cancer, and colorectal cancer.

**Results:**

The median (interquartile range) interval in days from first symptom presentation to the corresponding cancer diagnosis was: haematuria and urinary tract cancer, 59 (28–109); haemoptysis and lung cancer, 35 (18–89); dysphagia and gastro-oesophageal cancer, 25 (12–48); rectal bleeding and colorectal cancer, 49 (20–157). Three or more alarm symptom consultations were associated with increased odds of diagnosis of urinary tract cancer (odds ratio [OR] 1.84, 95% CI = 1.50 to 2.27), lung cancer (OR = 1.76, 95% CI = 1.07 to 2.90) and gastro-oesophageal cancer (OR = 2.17, 95% CI = 1.48 to 3.19). Longer diagnostic intervals were associated with increased mortality only for urinary tract cancer (hazard ratio 2.23, 95% CI = 1.35 to 3.69). Patients with no preceding alarm symptom had shorter survival from diagnosis of urinary tract, lung or colorectal cancer than those presenting with a relevant alarm symptom.

**Conclusion:**

After alarm symptom presentation, repeat consultations are associated with cancer diagnoses. Longer diagnostic intervals appeared to be associated with a worse prognosis for urinary tract cancer only. Mortality is higher when cancer is diagnosed in the absence of alarm symptoms.

## INTRODUCTION

Delayed cancer diagnosis is suggested to be a major reason for the observed poorer cancer survival outcomes in England compared to some other Western European countries.^1^,^2^ Early diagnosis of cancer in primary care may lead to better cancer treatment outcomes and improved survival. Dysphagia, haematuria, haemoptysis, and rectal bleeding are commonly seen in clinical practice and are recognised to be ‘alarm symptoms’ potentially predictive of cancer diagnoses.^3^–^8^

The predictive value of most alarm symptoms for cancer is low^3^ and these symptoms are frequently associated with benign conditions. Medical practitioners typically use individual approaches (such as medical knowledge, experience, threshold of uncertainty) to select the minority of patients requiring urgent attention from the majority who are likely to have self-limiting disorders.^9^ Consequently, delays may occur following the first presentation to primary care. These delays may be explained by the lack of specificity of alarm symptoms, their low predictive value for cancer, as well as organisational factors, including delays in gaining access to investigations or specialist advice.^10^ However, the potential relationship between alarm symptom presentation and cancer survival is unclear. Few population-based studies have evaluated the length of diagnostic intervals following alarm symptoms presentations of cancer and the association with outcome, in terms of cancer survival. One population-based study of bladder cancer in south-east England, found that the median delay from referral to first treatment was 48 days (interquartile range 27–84) but, paradoxically, patients treated with the shortest delay had the lowest survival.^11^ Referred to as the ‘waiting time paradox’, the lowest survival associated with the shortest delay has been attributed to confounding by indication (that is, patients in more advanced stages of the disease being prioritised for treatment).^12^

The present study investigated the delay between a patient first presenting with an alarm symptom and the diagnosis of a related cancer, and evaluated the association between the nature and timing of clinical presentation and survival following cancer diagnosis. The occurrence of relevant types of cancer in patients presenting with new onset haematuria, haemoptysis, dysphagia, or rectal bleeding was specifically investigated.

## METHOD

### Practice and patient selection

The UK Clinical Practice Research Database (CPRD) with linked Cancer Registry (CR) data was used to estimate the signal strength of alarm symptoms in the diagnosis of cancer.^9^ Data were extracted from the CPRD (formerly known as General Practice Research Database). The study included general practices in England that were eligible to be linked with CR data. Only those practices which continuously contributed acceptable research quality data to the CPRD during the period 1 January 2001 to 31 December 2007 were included. There were 158 eligible practices representing all practices that consented to CR data linkage. All registered patients were selected who had not been previously diagnosed with cancer, who had at least 12 months of follow-up prior to the start of observation on 1 January 2002, and were further eligible if they had no relevant alarm symptom or cancer diagnosis documented on or before 31 December 2001. The study population has been detailed in a previous publication.^13^

**How this fits in**

‘Alarm symptoms’, including haematuria, haemoptysis, dysphagia, or rectal bleeding, are thought to be predictive of a cancer diagnosis. The implications for cancer survival are unknown. This study analysed data from primary care records, linked to cancer registrations, to evaluate the association of alarm symptom presentation with cancer survival. Intervals from alarm symptom presentation to cancer diagnosis were generally of several weeks and were shortest for dysphagia. However, long diagnostic intervals were only associated with mortality for urinary tract cancer. Patients diagnosed with cancer without typical alarm symptom presentations generally had higher mortality than patients with a relevant alarm symptom. In general practice, repeat records of alarm symptoms are more likely to be followed by a cancer diagnosis. A minority of patients experience long intervals before cancer diagnosis. Such intervals were inconsistently associated with survival, with atypical presentations being more likely to be associated with a worse prognosis.

For the present study, all patients were identified who had either an alarm symptom or a relevant neoplasm recorded for the first time between 1 January 2002 and 31 December 2006, including: haematuria and/or a diagnosis of urinary tract neoplasms (including neoplasms of the urethra, bladder, ureter, or kidney but excluding neoplasms of the prostate and other reproductive organs); haemoptysis and/or a diagnosis of respiratory tract neoplasms; dysphagia and/or a diagnosis gastro-oesophageal neoplasms only; rectal bleeding and/or a diagnosis of colorectal neoplasms. In general, GPs are expected to record all alarm symptoms presented during the consultation. There were 267 participants excluded because of incomplete dates for diagnosis of either symptom or cancer; or when the first recorded date of symptom was after the cancer diagnosis; or the date of symptom or cancer diagnosis were after date of death; or the death date was not valid. This left 5524 participants for the analysis.

### Date of diagnosis

The date of first alarm symptom presentation was extracted from CPRD data. The earliest of the CR and CPRD records were used to ascertain the date of cancer diagnosis and date of death. The CR data available to this project only included the month and year of diagnosis, and a random day of diagnosis was imputed from a uniform distribution. When the CR date of cancer diagnosis was after the date of the alarm symptom in CPRD, the CPRD date of cancer diagnosis was used. Potential reasons for discrepancies in date of diagnosis between CR and CPRD include delay in documenting a cancer diagnosis in CPRD or erroneous documentation of an alarm symptom as a cancer event in CPRD.^13^ Sensitivity analyses ignoring cases where date of cancer diagnosis was prior to date of first alarm symptom presentation did not alter the study results.

### Analysis

For each patient, the time from the first recorded consultation with the relevant alarm symptom to the first diagnosis of the corresponding cancer of interest (interval to diagnosis), and the time from cancer diagnosis to date of death or the end of follow-up were calculated. Non-parametric rank sum tests were used to compare median differences in time to diagnosis between age groups, sex, and different deprivation quintiles. Logistic regression was used to estimate the association between the number of alarm symptom records (a single alarm symptom recorded in repeated consultations) prior to a first relevant cancer diagnosis and the odds of relevant cancer diagnosis after adjusting for age and sex. These analyses included all participants with the relevant alarm symptom. Participants were then divided into five groups according to the delay to diagnosis (<15 days, 15–90 days, 91–180 days, 181–365 days, and >365 days). For each of the cancers of interest the association of the interval to diagnosis and presence of a relevant alarm symptom on the survival time from cancer diagnosis to death were evaluated using the survival time functions in Stata (version 9). To estimate whether the presence of a relevant alarm symptom was associated with survival time from cancer diagnosis to death, participants were grouped into those with a relevant alarm symptom (0) or without any alarm symptom (1). The latter group includes patients without any alarm symptom consultation recorded prior to a relevant cancer diagnosis. Cox regression was used to evaluate the age and sex adjusted hazard ratio (HR) of each group of patients relative to those whose delay to diagnosis was between 15 and 90 days, as well as the HR of patients without an alarm symptom versus those with a relevant alarm symptom. The 15–90 days group was selected as the reference group because it included the largest proportion of patients resulting in smallest standard errors and narrower confidence intervals (CIs). The proportional hazards assumption was evaluated using the Schoenfeld residuals.

## RESULTS

Table 1 shows the numbers of patients who presented with a relevant alarm symptom and were diagnosed with a relevant cancer during the study period. The distribution of time intervals from first alarm symptom presentation to relevant cancer diagnosis, is shown for all participants and by age group and sex. The median time from first relevant alarm symptom presentation to relevant cancer diagnosis was 59 days for haematuria, 35 days for haemoptysis, 25 days for dysphagia, and 49 days for rectal bleeding. Time from first presentation to diagnosis did not vary systematically by age group or sex. However, for gastro-oesophageal cancer younger patients tended to show a slightly longer time (*P* = 0·065) between relevant symptom presentation and a relevant cancer diagnosis.

**Table 1.** Time interval in days from symptom presentation to diagnosis by age and sex

	**Urinary tract cancer**	**Lung cancer**	**Gastro-oesophageal cancer**	**Colorectal cancer**
Eligible,^a^ *n*	906	283	437	498

Excluded,^b^ *n* (%)	64 (7)	68 (24)	88 (20)	47 (9)

Included, *n* (%)	842 (93)	215 (76)	349 (80)	451 (91)

**Median time from symptom to diagnosis, days (IQR)**				

All subjects	59 (28–109)	35 (18–89)	25 (12–48)	49 (20–157)

**Age, years**				
≤69	56 (26–113)	35 (15–84)	27 (15–51)	52 (21–175)
>69	61 (31–107)	34 (18–91)	22 (10–42)	49 (18–125)
*P*-value	0.357	0.890	0.065	0.247

**Sex**				
Male	58 (29–117)	32 (19–102)	24 (10–44)	49 (19–129)
Female	59 (28–106)	38 (15–87)	25 (13–50)	49 (21–165)
*P*-value	0.825	0.959	0.565	0.584

a These figures include all patients with a relevant alarm symptom and relevant cancer diagnosis.

b Cases with incomplete dates for diagnosis of symptom or cancer, first symptom after cancer diagnosis, first symptom or cancer diagnosis after date of death, or no valid date of death were excluded. IQR = interquartile range.

Table 2 shows the number of consultations with a relevant alarm symptom recorded between first record of the alarm symptom and the relevant cancer diagnosis. The proportion of subjects with only one presentation for relevant alarm symptoms before cancer diagnosis was 55% for haematuria, 71% for haemoptysis, 69% for dysphagia, and 75% for rectal bleeding. The odds of relevant cancer diagnosis increased with the number of consultations with the relevant alarm symptom. Patients with three alarm symptom records had higher odds of urinary tract cancer (odds ratio [OR] = 1.84, 95% CI = 1.50 to 2.27), lung cancer (OR = 1.76, 95% CI = 1.07 to 2.90), or gastro-oesophageal cancer (OR = 2.17, 95% CI = 1.48 to 3.19). This pattern of association was not observed for colorectal cancer.

**Table 2.** Number of consultations for alarm symptoms before cancer diagnosis and odds of relevant cancer diagnosis. Odds ratios were adjusted for age and sex

**Consultations before diagnosis**	**Urinary tract (*n* = 842)**		**Lung cancer (*n* = 215)**		**Gastro-oesophageal (*n* = 349)**		**Colorectal (*n* = 451)**	
	***N* (%)**	**OR (95%CI)**	***N* (%)**	**OR (95%CI)**	***N* (%)**	**OR (95%CI)**	***N* (%)**	**OR (95%CI)**
One	453(55)	Ref	153(71)	Ref	242(69)	Ref	338(75)	Ref
Two	251(30)	1.97 (1.67 to 2.33)^a^	41(19)	1.37 (0.94 to 1.98)	73(21)	1.63 (1.24 to 2.15)^a^	87(19)	1.18 (0.93 to 1.51)
Three	138(15)	1.84 (1.50 to 2.27)^a^	21(10)	1.76 (1.07 to 2.90)^a^	34(10)	2.17 (1.48 to 3.19)^a^	26(6)	0.91 (0.60 to 1.37)

N = number. OR = odds ratios. Ref = reference category.

a Statistical significance at the 0.05 level.

Table 3 shows the association between survival from the time of cancer diagnosis and length of diagnostic interval before cancer diagnosis. For urinary tract cancer, patients with the longest interval (>365 days) from first haematuria presentation to cancer diagnosis had the highest mortality (HR = 2.22, 95% CI = 1.35 to 3.69) compared to patients with an interval duration of 15 to 90 days. No association was observed between survival from first cancer diagnosis to death for lung, gastro-oesophageal, and colorectal cancers by time interval before diagnosis (Table 3).

**Table 3.** Adjusted relative risk of mortality following diagnosis by duration of interval before diagnosis

	**Interval**	***n***	**Deaths**	**HR^a,b^**	**95% CI**	***P*-value**
Urinary tract (*n*= 842)	≤14	92	33	1.38	(0.84 to 2.27)	0.200
	15–90	479	144	Ref	–	
	91–180	151	42	1.10	(0.74 to 1.62)	0.246
	181–365	59	20	1.11	(0.64 to 1.93)	0.720
	>365	61	26	2.23	(1.35 to 3.69)	0.002^c^

Lung cancer (*n*= 215)	≤14	46	40	1.13	(0.75 to 1.71)	0.549
	15–90	116	104	Ref	–	
	91–180	23	17	1.14	(0.68 to 1.91)	0.622
	181–365	15	12	1.80	(0.86 to 3.79)	0.120
	>365	15	8	0.57	(0.26 to 1.26)	0.166

Gastro-oesophageal cancer (*n*= 349)	≤14	104	88	0.86	(0.64 to 1.16)	0.324
	15–90	209	172	Ref	–	
	91–180	20	16	0.77	(0.44 to 1.34)	0.357
	181–365	10	7	1.03	(0.29 to 3.65)	0.963
	>365	5	4	0.50	(0.23 to 1.07)	0.073

Colorectal cancer (*n*= 451)	≤14	79	26	1.07	(0.65 to 1.77)	0.794
	15–90	217	74	Ref	–	
	91–180	56	21	1.28	(0.73 to 2.25)	0.386
	181–365	44	12	0.76	(0.38 to 1.54)	0.448
	>365	55	12	0.92	(0.45 to 1.85)	0.806

a Interval, time from symptom to cancer diagnosis (days).

b Age and sex adjusted.

c Statistical significance at the 0.05 level. HR = hazard ratios for mortality following diagnosis date. N = number. Ref = reference category.

Regarding differences in survival from cancer diagnosis to death (Table 4), patients without any record alarm symptom presentation had higher mortality than patients with a relevant alarm symptom. Patients with urinary tract cancer who presented without any alarm symptoms had a relative hazard (HR) of 1.66 (95% CI = 1.28 to 2.15); for lung cancer HR = 1.29 (95% CI = 1.10 to 1.51), and for colorectal cancer HR = 1.74 (95% CI = 1.42 to 2.13). This pattern of association did not hold for gastro-oesophageal cancer.

**Table 4.** Adjusted relative risk of mortality by presence or absence of an alarm symptom

	***N***	**Deaths**	**HR^a^**	**95% CI**	***P*-value**
**Urinary tract**					
Haematuria symptom	842	265	Ref	–	
No haematuria	488	190	1.66	(1.28 to 2.15)	<0.001^b^

**Lung cancer**					
Haemoptysis symptom	215	161	Ref	–	
No haemoptysis	1380	1208	1.29	(1.10 to 1.51)	0.002^b^

**Gastro-oesophageal cancer**					
Dysphagia symptom	349	287	Ref	–	
No dysphagia	505	406	0.92	(0.78 to 1.09)	0.333

**Colorectal cancer**					
Rectal bleeding symptom	451	145	Ref	–	
No rectal bleeding	1294	650	1.74	(1.42 to 2.13)	<0.001^b^

a Age and sex adjusted.

b Statistical significance at the 0.05 level or the 0.001 level. HR = hazard ratios for mortality following diagnosis date. N = number. Ref = reference category.

## DISCUSSION

### Summary

These results provide population-based data for the time intervals between the presentation with an alarm symptom and the subsequent diagnosis with a related cancer. In general, the median intervals range from just under a month for dysphagia to around 2 months for haematuria. For cancers with short survival, the time from first alarm symptom presentation to relevant cancer diagnosis represents a large part of the overall survival from alarm symptom presentation to death. For each symptom–cancer pair, a wide range of time intervals were observed and it was found that for patients with urinary tract cancer those patients with longer delay in cancer diagnosis were those with the lowest survival from cancer diagnosis to death. In general, patients without any record of alarm symptom had lower survival from cancer diagnosis to death than those with a relevant alarm symptom.

### Strengths and limitations

The study is based on a large prospectively-collected, population-based data set enhancing representativeness of the findings. The use of prospectively collected data from primary care minimises the risk of selection, recall or responder bias. The authors have recently validated the date of diagnosis and date of death with respect to urinary tract, colorectal, gastro-oesophageal, and lung cancers in the CPRD using cancer registration data.^13^ The limitations are those typical of studies using routinely collected data. The CR data available to the study did not contain the day of cancer diagnosis (only year and month). However, the study’s ability to link CPRD with CR data minimised any bias associated with incomplete CR dates of diagnosis. Methodological concerns such as lead time and length bias are common problems with cancer survival analyses^14^ and the study is not immune to these biases. However, if cancer patients with alarm symptoms present earlier in their illness, then lead time bias could explain the difference in survival compared with patients who did not present with alarm symptoms. A number of policy initiatives on early diagnosis of cancer since 2008 may reduce the generalisability of some of the findings. For instance, widening rapid access to diagnostic procedures under the ‘2-week wait’ process, and increased awareness of both patients and doctors may have led to shorter diagnosis interval delay in recent years. However, the important finding of better prognosis associated with the presence of alarm symptoms may be generalisable to current practice and other populations. The study considered a single alarm symptom for each type of cancer, although they are the most commonly reported alarm symptoms in primary care for the types of cancer considered here.

### Comparison with existing literature

Previous studies of colorectal cancer,^12^,^15^ found slightly shorter diagnostic interval (40 days and 42 days, respectively) compared to this study (49 days). These studies included all colorectal cancer alarm symptoms rather than focusing on the specific alarm symptom of rectal bleeding which makes direct comparison difficult. These studies also used a much shorter study period (3 months and 1 year, respectively). In a systematic review, Ramos *et al*^16^ suggested no relationship between diagnostic delay and colorectal cancer survival, and this study’s findings are consistent with this suggestion. The higher survival for patients with a short delay from alarm symptom presentation to urinary tract cancer diagnosis is in line with findings of Wallace *et al.*^17^ The association between numbers of presentations with the same relevant alarm symptom and increased risk of a relevant cancer diagnosis is a novel finding that warrants additional investigation. If all patients with a relevant alarm symptom would be investigated at the first presentation the association between first ‘alarm presentation’ and cancer diagnosis may be stronger. Differences in survival between patients with or without a relevant alarm symptom is less explored and the findings indicate that this area is worthy of further investigation.

While unexpected the findings for the shortest survival among patients with the shortest diagnosis interval are consistent with previous studies.^12^,^18^ A plausible explanation for this finding is that patients in more advanced stages of the disease presented later with the alarm symptom, leading to worst survival. Diagnostic difficulties in slow growing tumours that are hard to detect could also explain the observed waiting time paradox.^19^

The findings of the study need also to be seen in the context of potential GP biases in coding of alarm symptoms. GPs may be more likely to code an alarm symptom as main symptom if they are not suspicious of a possible cancer event. The accuracy of alarm symptom coding would be higher when alarm symptom presentation results in a referral for confirmation of diagnosis. There may also be variation in patients’ health-seeking behaviour or GPs referral for diagnosis confirmation among different alarm symptoms. Patients and GPs, for instance, may be less worried about alarm symptoms with lower predictive value (that is, rectal bleeding) as opposed to those with higher predictive value (that is, haemoptysis).^9^

### Implications for research and practice

Currently there is ongoing debate about the value of alarm symptoms for early cancer diagnosis.^20^ The finding in this study of higher mortality among patients without an alarm symptom suggests that these symptoms may be important in drawing attention to an underlying cancer, leading to earlier diagnosis. However, there remains substantial variation in the time interval from first relevant alarm symptom presentation to relevant cancer diagnosis across these four common cancer sites. Patients who consult several times for alarm symptoms are more likely to be diagnosed with cancer. These observations suggest that clinical priority should be given to these symptoms, especially when they are persistent. This study showed an association between longer time intervals before diagnosis and cancer prognosis only for one cancer site. The authors caution that in an observational study, with incomplete information for important variables including cancer stage and grade, it would be inappropriate to conclude that a conservative approach to management of patients presenting with alarm symptoms would be justified.

## Open access

This is an Open Access article distributed in accordance with the CC BY-NC 3.0 license http://creativecommons.org/licenses/.
